# Incompatibility of lyophilized inactivated polio vaccine with liquid pentavalent whole-cell-pertussis-containing vaccine

**DOI:** 10.1016/j.vaccine.2016.07.030

**Published:** 2016-08-31

**Authors:** Heleen Kraan, Rimko ten Have, Larissa van der Maas, Gideon Kersten, Jean-Pierre Amorij

**Affiliations:** a Intravacc (Institute for Translational Vaccinology), Antonie van Leeuwenhoeklaan 9, P.O. Box 450, 3720 AL Bilthoven, The Netherlands; b Division of Drug Delivery Technology, Leiden Academic Center for Drug Research, Leiden University, Leiden, The Netherlands

**Keywords:** Inactivated polio vaccine, Thimerosal, Lyophilization, Rat potency, Hexavalent vaccine

## Abstract

A hexavalent vaccine containing diphtheria toxoid, tetanus toxoid, whole cell pertussis, *Haemophilius influenza* type B, hepatitis B and inactivated polio vaccine (IPV) may: (i) increase the efficiency of vaccination campaigns, (ii) reduce the number of injections thereby reducing needlestick injuries, and (iii) ensure better protection against pertussis as compared to vaccines containing acellular pertussis antigens. An approach to obtain a hexavalent vaccine might be reconstituting lyophilized polio vaccine (IPV-LYO) with liquid pentavalent vaccine just before intramuscular delivery. The potential limitations of this approach were investigated including thermostability of IPV as measured by D-antigen ELISA and rat potency, the compatibility of fluid and lyophilized IPV in combination with thimerosal and thimerosal containing hexavalent vaccine.

The rat potency of polio type 3 in IPV-LYO was 2 to 3-fold lower than standardized on the D-antigen content, suggesting an alteration of the polio type 3 D-antigen particle by lyophilization. Type 1 and 2 had unaffected antigenicity/immunogenicity ratios. Alteration of type 3 D-antigen could be detected by showing reduced thermostability at 45 °C compared to type 3 in non-lyophilized liquid controls.

Reconstituting IPV-LYO in the presence of thimerosal (TM) resulted in a fast temperature dependent loss of polio type 1-3 D-antigen. The presence of 0.005% TM reduced the D-antigen content by ∼20% (polio type 2/3) and ∼60% (polio type 1) in 6 h at 25 °C, which are WHO open vial policy conditions. At 37 °C, D-antigen was diminished even faster, suggesting that very fast, i.e., immediately after preparation, intramuscular delivery of the conceived hexavalent vaccine would not be a feasible option. Use of the TM-scavenger, l-cysteine, to bind TM (or mercury containing TM degradation products), resulted in a hexavalent vaccine mixture in which polio D-antigen was more stable.

## 1 Introduction

Combination vaccines are very successful, especially for delivery in children. The inclusion of multiple vaccine antigens in a single formulation reduces the number of injections, facilitates inclusion of new vaccines and increases coverage of routine pediatric immunization programs. For example, the use of pentavalent vaccine combining diphtheria-tetanus-pertussis (DTP), *Haemophilus influenzae* type B (Hib) and hepatitis B (HBV) antigens has raised the coverage of Hib and hepatitis B in the poorest developing (Gavi-supported) countries [1].

One of the challenges for an IPV-containing hexavalent vaccine is the presence of the preservative thimerosal (TM). TM negatively affects the antigenicity and immunogenicity of IPV [2] and is used in the production process of whole cell pertussis (wP) vaccine as an inactivating agent as well as a preservative [3]. Hence, pentavalent vaccine contains trace amounts of TM (⩽0.01% (w/v)).

Currently, the globally marketed IPV-containing hexavalent pediatric combination vaccines (Infanrix Hexa® (GSK) and Hexaxim® (Sanofi Pasteur)) contain an acellular pertussis (aP) component, which is devoid of TM. The use of wP in hexavalent vaccines intended for developing countries is important because of the lower costs and emerging doubts about the long-term effectiveness of aP vaccines. Unfortunately, no hexavalent combinations with wP (without TM) are licensed or in late-stage development [1].

The aim of this study is to investigate whether IPV-LYO, as previously developed [4], could be used in combination with a wP-containing pentavalent vaccine to generate a concept hexavalent vaccine, for example, for use in developing countries. By reconstituting IPV-LYO with pentavalent vaccine no substantial change in total volume is anticipated, likely the same injection volume for IM-injection may be used.

This study addresses the (thermo)stability of IPV-LYO with respect to both D-antigenicity and immunogenicity (rat potency) and shows D-antigenicity data on IPV-LYO reconstituted with a pentavalent vaccine (DTwP-Hib-HBV).

## 2 Materials and methods

### 2.1 Materials

The IPV used in this study is a ten times concentrated trivalent bulk containing the inactivated Mahoney (type 1), MEF (type 2) and Saukett (type 3) strains at a nominal D-antigen content (expressed in D-units, DU) of 400-80-320 DU/mL (for types 1, 2 and 3, respectively) and produced under cGMP conditions according to a routine production process [5]. The pentavalent vaccine, Diphtheria, Tetanus, (whole cell) Pertussis, Hepatitis B and *Heamophilus influenza* type b Conjugate Vaccine Adsorbed, was a gift from Serum Institute of India (SII).

D-sorbitol, magnesium chloride hexahydrate (MgCl_2_·6H_2_0) and monosodium glutamate monohydrate were from Sigma-Aldrich (St. Louis, MO). Citric acid (Sigma-Aldrich, St. Louis, MO) and disodium hydrogen phosphate (Fluka, Buchs, Switzerland) were used to prepare McIlvaine buffer. Thimerosal (TM) and l-cysteine were from Sigma-Aldrich (St. Louis, MO). All excipients used were of reagent quality or of a higher grade.

### 2.2 Methods

#### 2.2.1 Formulating IPV

Unless indicated otherwise, the trivalent IPV bulk material was dialyzed against 10 mM McIlvaine buffer (pH 7.0) using a low-binding regenerated cellulose membrane dialysis cassette (*M_w_* cut-off = 10 kDa). The dialyzed IPV was diluted 1:1 with formulation buffer containing: D-sorbitol (20% w/v), MgCl_2_·6H_2_0 (17% w/v), and monosodium glutamate monohydrate (17% w/v) in McIlvaine buffer (10 mM, pH 7.0). This formulated IPV was used for the preparation of IPV-LYO. Liquid IPV was prepared by 1:1 dilution of (not dialyzed) trivalent IPV with ultrapure water. This material was used as a control in experiments.

#### 2.2.2 Lyophilization

Injection vials (3 mL, Aluglas BV, Uithoorn, The Netherlands) were filled with 0.2 mL of the formulated IPV and half-stoppered with pre-dried (overnight at 105 °C) 13 mm lyophilization stoppers (PH21/50 from Aluglas BV, Uithoorn, The Netherlands). Vials were loaded on precooled shelves (−50 °C) and the solidified material was subsequently lyophilized. Primary drying was done at 0.045 mbar and −45 °C for 26 h. Secondary drying was done at a pressure of 0.01 mbar and shelf temperature that increased from −45 °C to 25 °C in 13.3 h. Thereafter, both shelf temperature (25 °C) and pressure (0.01 mbar) were kept constant for 24 h. After lyophilization, vials were closed under vacuum, sealed with alu-caps and stored for stability testing.

#### 2.2.3 Stability testing

For stability studies, liquid IPV (0.2 mL in stoppered and capped 3 mL injection vials) and IPV-LYO (in stoppered and capped 3 mL vials) were incubated at 2-8, 25, 37, and 45 °C. After various periods of time, vials were taken for analysis.

#### 2.2.4 Effect of thimerosal

The effect of thimerosal (TM) on liquid IPV was studied by diluting trivalent IPV 10-fold with a solution of TM in ultrapure water. IPV-LYO was reconstituted either with ultrapure water (0.5 mL), 0.5 mL TM solution, or 0.5 mL pentavalent SII-vaccine containing 0.005% (w/v) TM. Final TM concentrations were 0.005 and 0.01% (w/v).

The possible neutralizing effect of l-cysteine on TM was investigated by pre-incubating pentavalent vaccine for one hour with 0.05% (w/v) l-cysteine or ultrapure water as negative control. Subsequently, IPV-LYO was reconstituted with the pre-incubated pentavalent vaccines or with ultrapure water as a control. D-antigen recoveries were determined directly after mixing or after subsequent storage at 37 °C for 24 h.

#### 2.2.5 Analysis

##### 2.2.5.1 D-antigen ELISA

The D-antigen ELISA was performed as described elsewhere [6]. Microtiter plates were coated with serotype-specific bovine anti-polio serum (Bilthoven Biologicals, Bilthoven, The Netherlands). After washing, dilutions of IPV (reconstituted IPV-LYO or liquid) were added. After an incubation period of 30 min at 37 °C under gentle shaking, plates were washed. Subsequently, a mixture of serotype-specific anti-poliovirus monoclonal antibody (3-4E4, 3-14-4 and 1-12-9 for type 1, 2 and 3, respectively) and HRP-labeled anti-mouse IgG (GE Healthcare, Buckinghamshire, UK) was added and plates were incubated for 30 min. at 37 °C while shaking. Subsequently, plates were washed followed by addition of ELISA HighLight signal reagent (ZomerBloemen BV, Zeist, The Netherlands). Chemiluminescence was measured for 10–15 min by using a luminometer (Berthold Centro LB960). The signal at maximum intensity was used to calculate the D-antigen content relative to the reference standard.

##### 2.2.5.2 Biosensor analysis

Antigenicity was also measured using a Biacore T200 (GE Healthcare, Hoevelaken, The Netherlands), equipped with an anti-polio biosensor as described elsewhere [7]. Goat anti-mouse IgG Fc-specific (Thermo Fisher Scientific Inc, Waltham, MA), antibodies were covalently immobilized on the dextran layer of a CM3 sensorchip (GE Healthcare, Hoevelaken, The Netherlands) by primary amine coupling, following the manufacturers recommendations (GE Healthcare, Hoevelaken, The Netherlands). Serotype-specific monoclonal antibodies (3-4E4 (antigenic site 1, type 1), 3-14-4 (antigenic site 1, type 2), Hyb300-06 (antigenic site 1, type 3) and 1-12-9 (antigenic site 2/3/4, type 3) were bound to the sensor, followed by IPV. The sensor chip was regenerated with 10 mM glycine-HCl (pH 1.5). Assay data were analyzed by four-parameter curve fitting using the Biacore T200 evaluation software. Antigenicity was calculated relative to the international reference PU91-01.

##### 2.2.5.3 Rat potency

Immunogenicity of IPV-LYO was measured in the rat potency test performed as described earlier [7] with the exception that the highest dilution of the vaccine was not included. Animal experiments were conducted in accordance with the guidelines provided by the Dutch Animal Protection Act, and were approved by the Committee for Animal Experimentation of Intravacc. RIVM-TOX rats were immunized with four threefold dilutions of reconstituted IPV-LYO, the liquid IPV control, and the reference vaccine (PU91-01). After three weeks, sera were collected and neutralizing antibodies against all three poliovirus types were measured separately by inoculating Vero cells with 100 TCID_50_ of the wild-type strains (Mahoney, MEF-1 and Saukett) as described previously [8]. Two-fold serial serum dilutions were made and serum/virus mixtures were incubated for three hours at 36 °C and 5% CO_2_ followed by overnight incubation at 5 °C. Subsequently, Vero cells were added and after 7 days of incubation at 36 °C and 5% CO_2_ the plates were stained and fixed with crystal violet and the results were read macroscopically. Virus-neutralizing (VN) titers were expressed as the last serum dilution that has an intact monolayer (no signs of cytopathogenic effect). Immunogenicity was expressed in two ways: (A) as the relative potency to the reference vaccine using the parallel-line model, and (B) as the average virus-neutralizing antibody endpoint titer at the second (1/15 dilution) or third (1/45 dilution) highest vaccine dose.

##### 2.2.5.4 Moisture content analysis

Residual moisture content was determined using a Karl Fischer Coulometer C30 (Mettler-Toledo, Tiel, The Netherlands) according to the literature [4]. Samples were weighed, reconstituted in Hydranal Coulomat A (Fluka, Buchs, Switzerland), and injected into the titration vessel. The empty vials were weighed and water content was calculated based on the measured water content, the weight of the lyophilized product in the vial, the reconstitution volume of the reagent, the titration volume, and the water content of the blank titration.

##### 2.2.5.5 Statistical analysis

For comparative analysis of immunogenicity, data were tested by one-way analysis of variance (ANOVA) followed by a Bonferonni test for multiple comparisons. Probability (*p*) values <0.05 were considered significant. All statistical analyses were performed using GraphPad Prism version 6.0 (GraphPad Software Inc., La Jolla, CA).

## 3 Results

### 3.1 Immunogenicity of IPV-LYO

**Fig. 1:**
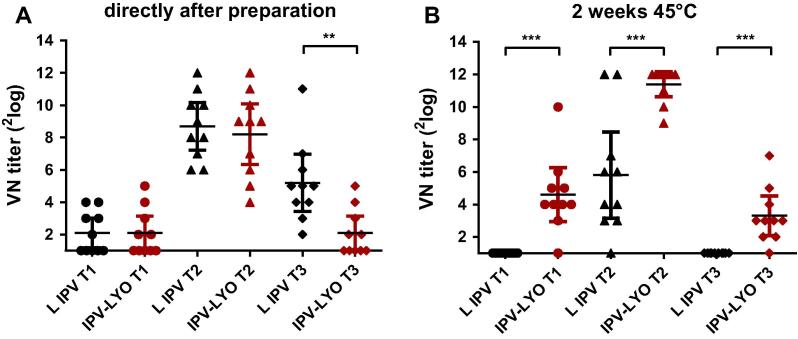
Mean virus-neutralizing (VN) titers of serum from rats (*n* = 10) immunized with 1/45 human dose (panel A) or 1/15 human dose (panel B) of liquid IPV (L IPV, in black) or IPV-LYO (in red) directly after preparation or after subsequent two weeks storage at 45 °C. Individual VN titers specific for serotype 1 (circles), 2 (triangles) and 3 (diamonds) were shown. Mean values were depicted as horizontal line and error bars showed 95% confidence interval (CI) values. Asterisks indicate significant differences between groups (^*^p < 0.01, ^**^p < 0.001, ^***^p < 0.0001).

Two batches of IPV-LYO were prepared, characterized and used for further experiments. The D-antigen composition, D-antigen recovery, and RMC (see Table 1), and thermostability (data not shown) of both batches were comparable. Immediately after lyophilization, the rat potency of IPV-LYO was 0.88, 1.17 and 0.39, respectively for polio type 1, 2 and 3. The rat potency of type 3 was lower than anticipated as based on: (A) the D-antigen concentration, and (B) historical data of polio containing vaccines (data not shown). Virus-neutralizing (VN) titers of polio type 3 were significantly lower in rats immunized with IPV-LYO if compared to liquid IPV (see Fig. 1A). This unanticipated result was confirmed with an independent IPV-LYO preparation and throughout subsequent experiments (see Section 3.2).

In an earlier study it was demonstrated that disruption of antigenic site 1 diminished the rat potency of polio type 3 [9]. However, Biosensor analysis showed that antigenic site 1 remained intact after freeze-drying, as observed D-antigen concentrations were 130 ± 5 DU/mL (IPV-LYO) and 137 ± 1 DU/mL (liquid IPV).

### 3.2 Stability of IPV-LYO

Accelerated stability testing for two weeks at 45 °C, showed significantly higher VN-titers for all three serotypes in IPV-LYO when compared to liquid IPV (Fig. 1B), the low average VN-titer of type 3 in IPV-LYO was retained, whereas it was completely nullified in liquid IPV.

**Fig. 2:**
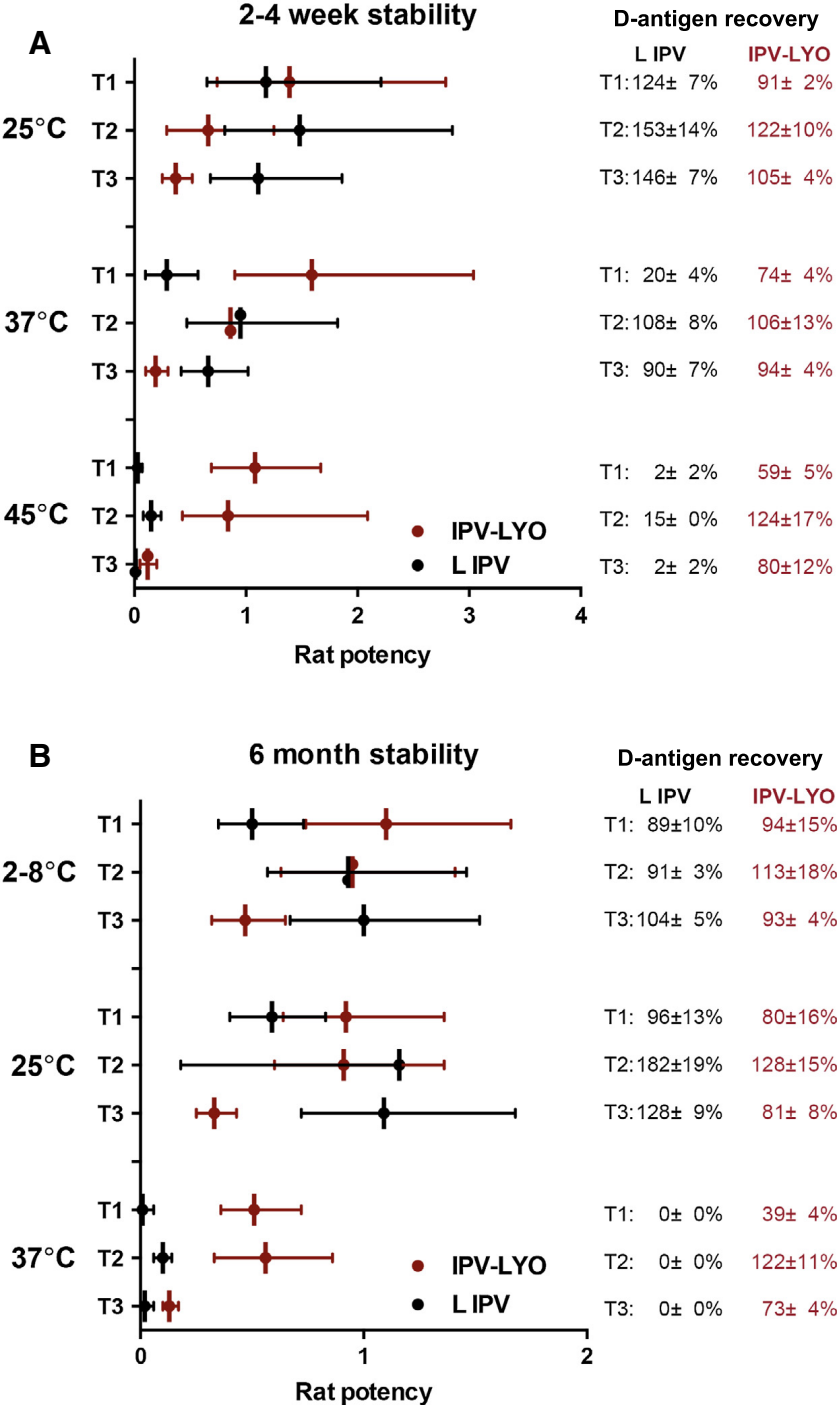
Rat potency of liquid IPV (L IPV) and IPV-LYO after incubation for a short period of time; 2 weeks at 45 °C or one month at 25 °C or 37 °C (panel A), or long period of time (6 months at 4 °C, 25 °C or 37 °C) (panel B). The rat potency is calculated based on a theoretical composition of 40, 8, 32 DU for type 1, 2, and 3, respectively.

In general, at temperatures of 37 °C and 45 °C, the recovery of type 1-3 D-antigen was lower in liquid IPV than in IPV-LYO (see Fig. 2). After four weeks storage at 25 °C, the rat potency of liquid IPV was approximately 1 for all serotypes, whereas significantly lower potency values were observed after short-term storage at 37 °C or 45 °C (see Fig. 2A). In contrast, IPV-LYO maintained its potency after short-term storage at 25–45 °C.

Even after storage for up to 6 months at 2–8 °C or 25 °C, no further decrease in potency was observed in case of IPV-LYO (see Fig. 2B).

During 6 months of storage at 37 °C, liquid IPV showed no measurable D-antigen recovery and the rat potency was almost completely lost, whereas IPV-LYO still showed D-antigen recoveries of 39 ± 4%, 122 ± 11% and 73 ± 4%, respectively for type 1, 2 and 3, and significantly higher rat potency values than liquid IPV (see Fig. 2B).

The rat potency of polio type 3 in IPV-LYO was consistently 2 to 3-fold lower than anticipated as based on the recovered D-antigen percentage (see Fig. 2A and B).

### 3.3 Thermostability of polio type 3 in reconstituted IPV-LYO

To examine whether IPV-LYO (type 3) after reconstitution was more vulnerable to higher temperatures than liquid IPV, a thermostability experiment was performed using reconstituted IPV-LYO, and both formulated liquid IPV and liquid IPV as controls.

**Fig. 3:**
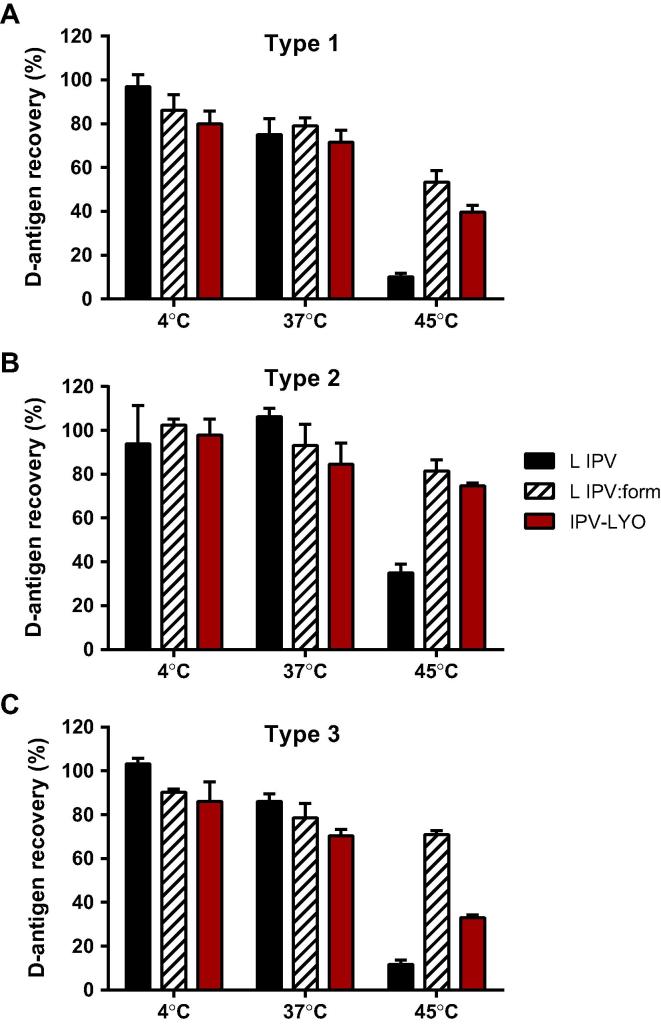
Stability of reconstituted IPV-LYO. Liquid IPV (L IPV, black bars), formulated (liquid) IPV prior to lyophilization (L IPV:form, striped bars), and reconstituted IPV-LYO (red bars) were incubated for 24 h at 4 °C, 37 °C or 45 °C. Subsequently, D-antigen recoveries were determined by ELISA, specific for type 1 (panel A), type 2 (panel B) and type 3 (panel C), and normalized for D-antigen recoveries directly after lyophilization. Mean values (*n* = 3) and SD are shown.

An incubation period of 24 h at either 4 °C or 37 °C did not result in a difference between the samples (see Fig. 3A–C). However, at 45 °C the D-antigen recovery of liquid IPV was clearly lower than the formulated liquid IPV, and resuspended IPV-LYO. From Fig. 3C it is clear that freeze-drying and resuspension rendered a type 3 particle characterized by less thermoresistance (recovery 33%) than its formulated counterpart (recovery 71%). Such a difference was not observed in case of polio serotype 1 and 2 (see Fig. 3A and B). From these findings it is hypothesized that the type 3 particle was altered by the lyophilization process rendering type 3 with a lower thermostability and a lower specific immunogenicity.

### 3.4 Effect of thimerosal containing pentavalent vaccine on IPV-LYO

**Fig. 4:**
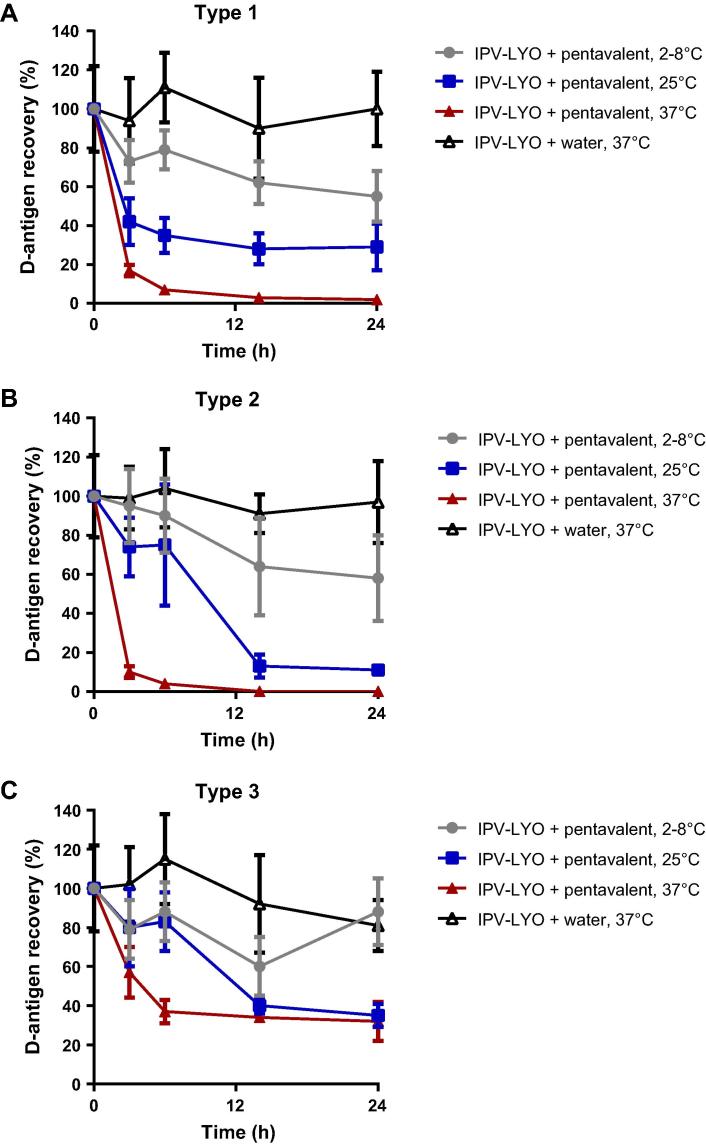
Effect of pentavalent vaccine on IPV-LYO. IPV-LYO was reconstituted with pentavalent vaccine containing 0.005% of thimerosal at temperatures of 2-8 °C (closed circles, grey), 25 °C (closed squares, blue) or 37 °C (closed triangles, red) for up to 24 h. Subsequently, D-antigen recoveries were determined by ELISA, specific for type 1 (panel A), type 2 (panel B) and type 3 (panel C). Mean values (*n* = 3) and SD are shown.

Reconstitution of IPV-LYO with pentavalent vaccine (0.005% thimerosal, TM) resulted in an evident negative trend in D-antigen recovery (see Fig. 4). The control, IPV-LYO reconstituted with ultrapure water, showed either no (in case of type 1 and 2) or a minimal loss (in case of type 3) in D-antigen recovery during incubation for 24 h at 37 °C.

The negative effect of TM on polio D-antigen was increased at a higher temperature in the range from 2–8 °C to 37 °C. After 6 h at 25 °C (relevant conditions for the WHO open vial policy), there was already a marked (and unacceptable) loss of ∼20% in case of type 2 and 3 and ∼60% in case of type 1.

A similar drop in D-antigenicity was observed when incubating IPV-LYO with 0.005% TM solution (data not shown) indicating that the negative effect on D-antigen recovery of IPV-LYO was most likely caused by the presence of TM.

### 3.5 Effect of pentavalent vaccine on IPV-LYO after pre-incubation with l-cysteine

**Fig. 5:**
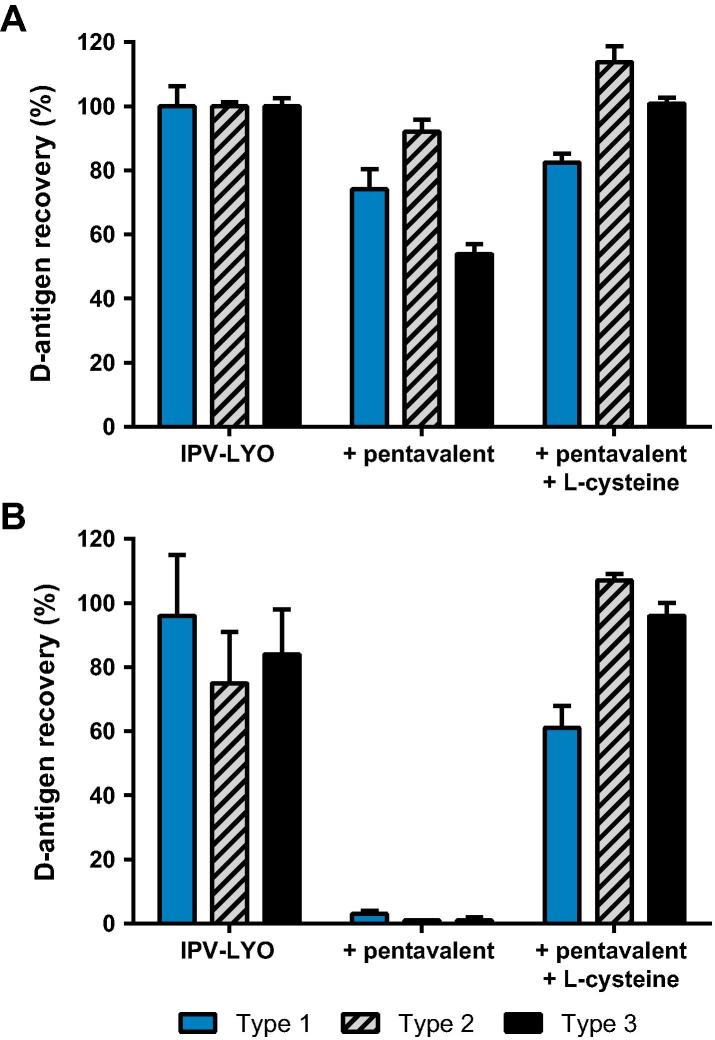
Effect of cysteine on the stability of IPV-LYO mixed with pentavalent vaccine. IPV-LYO was mixed with pentavalent vaccine pre-incubated for one hour in the absence or presence of l-cysteine. Subsequently, D-antigen recoveries directly after mixing (panel A) and after 24 h incubation at 37 °C (panel B) were determined by ELISA, specific for type 1 (black bars), type 2 (striped bars) and type 3 (red bars), and normalized for D-antigen recoveries of IPV-LYO prior to mixing and incubation. Mean values (*n* = 3) and SD are shown.

Low molecular weight thiols are well known for the ability to scavenge mercury and/or mercury containing compounds such as TM [10], [11]. Therefore, pentavalent vaccine was pre-incubated with the TM-scavenger l-cysteine and was used thereafter to reconstitute IPV-LYO. Fig. 5A shows a small initial loss of the D-antigen recovery upon reconstituting IPV-LYO with pentavalent vaccine. This initial loss is absent in samples that are reconstituted with purified water or in samples in which TM was scavenged.

After 24 h at 37 °C there is almost no recoverable D-antigen in case pentavalent vaccine was used to resuspend IPV-LYO (see Fig. 5B). However, when IPV-LYO was reconstituted with l-cysteine containing pentavalent vaccine, D-antigen could be clearly be recovered, values were 61 ± 7%, 107 ± 2% and 96 ± 4% for type 1, 2 and 3, respectively.

## 4 Discussion

### 4.1 Detrimental effect of lyophilization on the rat potency of polio type 3

For release of inactivated polio vaccines two assays are available: an ELISA for determining the D-antigen concentration (antigenicity) and the rat potency test for determining the relative rat potency (immunogenicity). The European Pharmacopoeia (EP) allows to omit the rat potency test and to rely exclusively on the more accurate D-antigen ELISA. Hence, there should be a relationship between the *in vitro* and *in vivo* test, despite the considerable test variation in the rat potency test. Remarkably, in our study the *in vitro* test content was not predictable for the immunogenicity of IPV-LYO type 3 (contrary to the normal *in vitro* – *in vivo* relationship observed with type 1 and 2). The type 3 rat potency in IPV-LYO was 2 to 3-fold lower than anticipated as based on the measured D-antigen concentration. Earlier studies reported significant lower virus-neutralizing titers against type 3 in rats immunized with lyophilized IPV formulated in dissolvable mini-implants (bioneedles) [12] or weaker serological responses to IPV type 3 after microneedle patch vaccination of rhesus macaques compared to intramuscular injection [13].

The use of certain site-specific monoclonal antibodies in the ELISA is critical for the antigenicity-immunogenicity relation [9]. In the case of detection of polio serotype 3, it has been recommended to include measurement of antigenic site 1, because disruption of this site strongly diminishes the rat potency [9]. No evidence for antigenic site 1 disruption was observed in this study. However, by performing accelerated stability testing it was revealed that freeze-drying and reconstitution reduced the thermostability of polio type 3 and did not affect the thermostability of type 1 and 2.

The information on the thermostability and the rat potency of polio type 3 taken together suggested that lyophilization resulted in an altered type 3 particle. The nature of this particle remains speculative (an intermediate in the transition between D-antigen and C-antigen).

This study shows that accelerated stability might be of use to obtain a more reliable prediction of the rat potency of dried IPV in general.

Interestingly, improved thermostability was observed in formulated liquid IPV compared to conventional liquid IPV without extra excipients. This indicates that it is possible to stabilize polio D-antigen in the liquid state by the addition of excipients.

### 4.2 Use of IPV-LYO in a hexavalent vaccine: overcoming the detrimental effect of thimerosal

The current study investigated the possible use of lyophilized IPV as a component for mixing with a pentavalent vaccine (DTwP-Hib-HBV) thereby forming a hexavalent mixture. These licensed pentavalent vaccines all contain traces of thimerosal (TM), an organomercury compound known for its antiseptic and antifungal properties. Unfortunately TM may also negatively affect the antigenicity and immunogenicity of IPV [2]. Within hours after reconstitution of IPV-LYO with pentavalent vaccine (containing 0.005% TM), a strong reduction in D-antigenicity was observed. The antigenicity decrease was even more evident when the vaccine was kept at 37 °C showing that also immediate IM-delivery, after mixing IPV-LYO and pentavalent vaccine, is probably not a feasible option to mitigate the negative impact of TM on polio D-antigen, which is consistent with reported findings [2].

Pre-incubation of the TM-containing pentavalent vaccine with l-cysteine reduced the negative effect of TM on D-antigenicity of IPV-LYO after reconstitution with pentavalent vaccine. The use of l-cysteine for neutralization of TM in a pentavalent vaccine has not been published before. However, the use of l-cysteine as a TM-scavenger is well-known [10], [11], [14]. The use of compounds, which compete with polio D-antigen for binding to TM or stabilize IPV, might offer a potential step towards a TM-resistant IPV formulation (mix-and-shoot approach), which can be used as a component in a hexavalent vaccine. On the other hand, removing TM completely from the vaccine may be considered as well by, for example, reformulating wP (prior to using it for preparing a pentavalent mixture).

## 5 Conclusion

This research demonstrated a clear difference in rat potency between IPV-LYO and liquid IPV serotype 3. The reduced thermostability of type 3 in IPV-LYO after reconstitution suggested the formation of an altered type 3 particle during the lyophilization process.

Use of IPV-LYO as a component in a hexavalent vaccine by mixing with licensed pentavalent vaccine (DTwP-Hib-HBV) requires neutralization of TM, by for example l-cysteine, to avoid the damaging effect on polio D-antigen (mix-and-shoot approach).

Further formulation development is needed, in which screening based on D-antigen thermostability is included, to improve the potency of IPV-LYO type 3.

## Figures and Tables

**Table 1:** Characterization of two IPV-LYO batches that were used for further stability testing. D-antigen composition (DU/mL), D-antigen recovery and residual moisture content (RMC) of both batches were determined. Mean values and SD are shown (*n* = 3). Rat potency of batch 2 was determined as well. Relative rat potency values and lower and upper limits (95% confidence intervals (CI)) are shown.

IPV-LYO	RMC (%)	Type 1	Type 2	Type 3
**Batch 1**	8.9 ± 0.4			
Composition (DU/mL)	–	180 ± 16	37 ± 5	138 ± 5
D-antigen recovery (%)	–	83 ± 7	78 ± 10	75 ± 3

**Batch 2**	8.0 ± 0.3			
Composition (DU/mL)	–	155 ± 2	34 ± 2	126 ± 4
D-antigen recovery (%)	–	80 ± 1	86 ± 4	77 ± 3
Rat potency [95% CI]	–	0.88 [0.58–1.32]	1.17 [0.76–1.81]	0.39 [0.30–0.51]
